# MAKE-IT—A Lightweight Mutual Authentication and Key Exchange Protocol for Industrial Internet of Things

**DOI:** 10.3390/s20185166

**Published:** 2020-09-10

**Authors:** Karanjeet Choudhary, Gurjot Singh Gaba, Ismail Butun, Pardeep Kumar

**Affiliations:** 1 School of Electronics and Electrical Engineering, Lovely Professional University, Punjab 144411, India; karanchoudhary8399@gmail.com; 2 Department of Computer Science and Engineering, Chalmers University of Technology, SE-412 96 Gothenburg, Sweden; 3 Department of Computer Science, Swansea University, Swansea, Wales SA1 8EN, UK; pardeep.kumar@swansea.ac.uk

**Keywords:** authentication, industrial internet of things (IIoT), industry 4.0, protocol, security

## Abstract

Continuous development of the Industrial Internet of Things (IIoT) has opened up enormous opportunities for the engineers to enhance the efficiency of the machines. Despite the development, many industry administrators still fear to use Internet for operating their machines due to untrusted nature of the communication channel. The utilization of internet for managing industrial operations can be widespread adopted if the authentication of the entities are performed and trust is ensured. The traditional schemes with their inherent security issues and other complexities, cannot be directly deployed to resource constrained network devices. Therefore, we have proposed a strong mutual authentication and secret key exchange protocol to address the vulnerabilities of the existing schemes. We have used various cryptography operations such as hashing, ciphering, and so forth, for providing secure mutual authentication and secret key exchange between different entities to restrict unauthorized access. Performance and security analysis clearly demonstrates that the proposed work is energy efficient (computation and communication inexpensive) and more robust against the attacks in comparison to the traditional schemes.

## 1. Introduction

**Figure 1:**
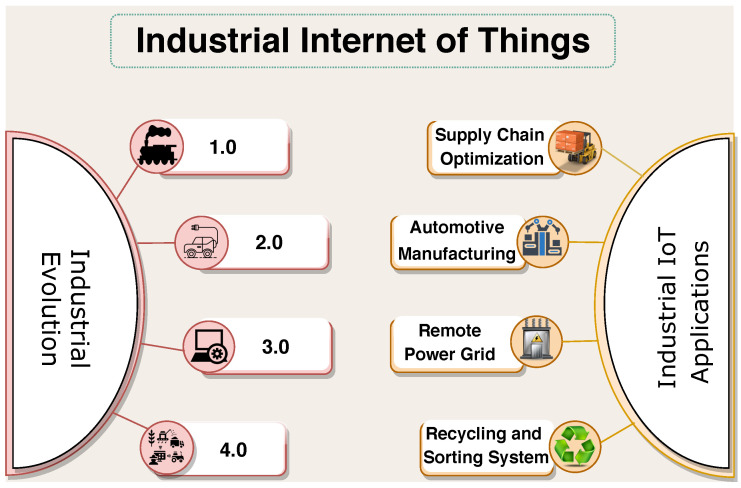
Industrial transformation and various applications of Industrial Internet of Things (IIoT).

The Industrial Internet of Things (IIoT) alias Industry 4.0 is the new era of the industrial revolution which uses the sensor and actuators for the enhancement of the production and manufacturing industrial process. IIoT is the 4th generation of the industrial development. The first generation (1.0) industries of 18th century made use of steam power to generate resources for their industry. The next revolution took place is industry 2.0 in the year 1870 where industries ran through electricity and assembly lines. Second revolution brought attention of engineers towards industrial development. The 3rd progress (3.0) in industrial development escalated the efficiency to a par level. It introduced the concept of computer and Programmable Logic Control (PLC) which was believed to be the first step towards automation of industries. The recent advancement of industry 4.0 uses additional infrastructure to connect industrial processes with the internet; thus permitting the engineers to control the machines remotely as well as allowing them to get instant access of the information through cloud storage [1]. The whole era of industrial transformation along with the various significant applications of IIoT is depicted in Figure 1.

IIoT is converging many traditional practices into intelligent and smart processes, few of the applications are illustrated in Figure 1 like supply chain optimization in warehouses, automotive manufacturing in industries, remote power generation monitoring and control in smart grids, recycling and sorting of waste products, and so forth. Another reason of motivation to industry owners for revamping their industries is the advancement in the field of micro electronics and ICT (Information & Communication Technology). The fundamental aim behind the evolution of IIoT is Machine to Machine (M2M) communication without human intervention [2]. M2M communication makes use of various equipment’s such as radio frequency identification (RFID), sensors, mobile devices, and wireless sensor networks (WSN) to achieve automation and seamless connectivity with other devices [3,4]. In addition, Internet of Things (IoT) enabled the industrial machines to upload the data on clouds for quick analyzing and decision making; thus eliminating the need of physical entries and analysis [5].

**Figure 2:**
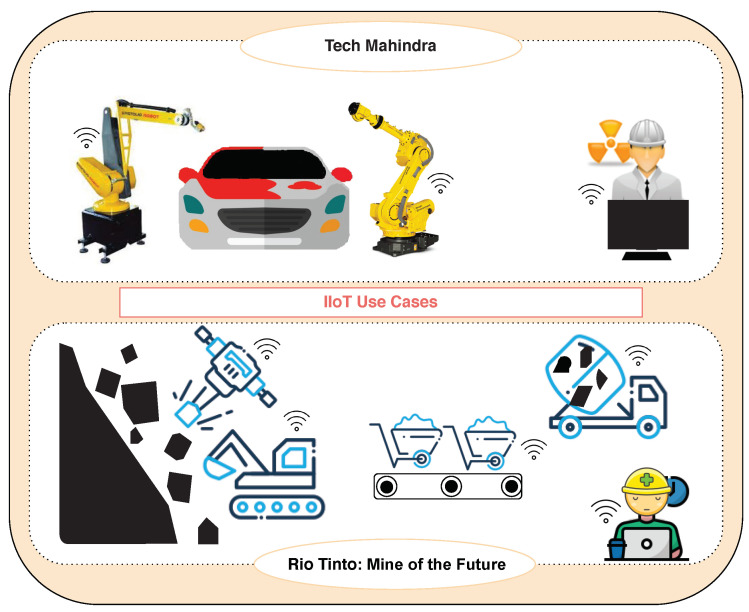
Few use cases of IIoT.

Industries with large manufacturing units are widespread adopting IIoT and have started making their machines IoT enabled. Some of the recent examples of its adoption are shown in Figure 2. Tech Mahindra (TM) is using IoT for monitoring and controlling painting and logistics section (transferring vehicle from production to manufacturing house). The use of IoT in TM enabled the workers to view the status of the assets anytime and anywhere. In addition, IoT strengthened the equipment and process diagnostic capabilities to reduce the time requirement from production to manufacturing [6]. Another application is implemented in Rio Tinto: Mine of the Future, the British and Australian mining industry [7]. They have launched an innovative automated mining machine in Pilbara, a remote region of Western Australia with the deep reserves of iron ore. The Driver less trucks and trains haul ore away from the mining sites while an autonomous drill technology enables a remote worker to oversee status of multiple drills from a single console. The company has a control center complex in Perth that connects to its mines as well as its rail and port operations, where the programmers, engineers, technicians, and analysts are remotely monitoring and guiding mining operations [7]. IIoT is a boon to industry owners as they can remotely visualize the performance of the staff, machines along with the status of the ongoing projects [8,9].

Modern ambush on cyber physical networks and systems upraise a solid security anxiety as such attacks can cause loss to customers, service providers, developers, and manufacturers [10,11]. The unknown vulnerabilities in the system like bugs, and broken processes, and so forth pave way to cyber attacks. Cyber attacks may result in loss of data privacy and integrity, illegal access to privileged zone, financial loss, and business disruption, and so forth. Inadequate or inappropriate security measures in IIoT can even lead to collapse of the whole industrial system. The setback of the industry operators happened due to news of attacks on IIoT networks: a network was created for controlling lights, fan, fire detection, and heating, ventilation, and air conditioning (HVAC) at Sochi arena for Olympics. But during inspection in 2018, it is found that 17,823 building automation control network (BACnet) devices and 78,000 supervisory control and data acquisition (SCADA) devices were exposed to internet without security protections. During investigation, the prime reason found is vulnerability in mutual authentication and key exchange protocol, that led the attackers to exploit the network resources [12].

*Forbes* reported an incident where attackers used malicious programs and communicating devices to harness the industrial network illegally. Attackers took over the charge of excavators, scrapers, and cranes, and so forth from the legitimate managers of the firm [13]. Another incident is informed by the security analyst firm, Zimperium Inc., USA. According to their report, IoT enabled electric scooter manufactured by Xiaomi Inc., China was accepting control commands for example, locking, braking, and acceleration, and so forth from even illegitimate users [13].

As thoroughly discussed in Reference [14], many IoT networks do not even possess basic security elements. On average, these are the cyber-security analysis of today’s COTS IoT products: 25 vulnerabilities are detected per device, 60% have vulnerable firmware, 70% do not encrypt any communications at all, and 80% fail to request a password for authentication that has a secure length.

There are few ways to protect IIoT against intrusions and cyber-attacks. One of them is allowing intrusions to happen and then detecting them via Intrusion Detection Systems [15], as discussed in Reference [16]. Alternatively, robust mutual authentication and secure key exchange procedures can be used to protect IIoT against attacks [17,18]. This article aims at providing a remedy by proposing: *A Lightweight **M**utual **A**uthentication and **K**ey **E**xchange Protocol for Industrial **I**nternet of **T**hings (MAKE-IT)*.

The motivation behind the proposed work is the limitations of the existing mutual authentication and key exchange protocols. The computation, message exchange, and communication cost of conventional protocols are large enough to drain the resources of the IoT constrained devices. These factors pave way for new mutual authentication and key exchange protocols that satisfy the requirements (robust and lightweight) of IoT networks.

MAKE-IT protocol protects the unauthorised access to industrial network through secure mutual authentication and key exchange process. Data confidentiality and integrity, to name a few, are ensured throughout the mutual authentication and key exchange process. MAKE-IT has significantly optimized the computation and communication processes in comparison to traditional protocols. Our network consideration covers industrial network settings in which private/off-the-grid network implementations are elected such as SCADA, Device Language Message Specification (DLMS)/Companion Specification for Energy Metering (COSEM), Modbus, and so forth. Here in our proposal, Authentication Server acts as a gateway in between the trusted industrial network site and un-trusted outside world. Therefore, users are treated as potential threats and authentication credentials are verified accordingly. Other than IIoT, our proposal might also be applicable to very specific subset of IoT, such as home automation systems where outside world connects the inside network via dedicated/trusted gateways. In this article, we have concentrated on private networks that are operating at industrial sites. This might be considered as a subset of IIoT in which the network is considered as secure, whereas end-users (operators, foremen, engineers, etc.) that are trying to read/write/execute commands at the industrial devices are considered to have potential threat to the industrial network due to various reasons: wireless communications, various attack landscape (Man-in-the-middle attack alias MITM attack, impersonation, etc.).

The remaining paper is structured as follows: Section 2 discusses the security protocols developed by peers. Section 3 presents the system and adversary model, and Section 4 describes the proposed scheme. Section 5 provides the formal and informal security analysis whereas Section 6 discusses the performance and comparative analysis. Section 7 draws the conclusions.

## 2. Related Work

Esfahani et al. [2] have discussed the role of Machine to Machine (M2M) communication in implementation of IIoT networks. As the nodes in the IIoT are resource constrained, therefore authors only utilized XOR and hashing operations to build the algorithm of authentication. The authors declared that their scheme provisions mutual authentication, confidentiality, session key, and so forth and is also resilient to replay, impersonation, and modification attacks, and so forth. The scheme proposed is simple yet secure but the communication, message exchange, and computational cost is large enough to drain the resources of the IoT constrained devices. Hence it may pose as a hindrance to the networks with sensitive resource constrained devices.

Li et al. [3] emphasized on the need and challenges of security in IoT due to unsecured open wireless channel and resource constrained devices. The authors suggested a three factor user authentication protocol to combat against legitimacy threats. The proposed protocol is claimed to be energy efficient and resistant to replay, impersonation attacks, and so forth. Xu et al. [19] have also disclosed a scheme for authentication of multi gateway wireless sensor networks connected with IoT based on 3 factor mechanism. Formal analysis using ProVerif proved that scheme is resistant against many potential attacks. However, as per Reference [20], both the protocols drain a significant amount of precious energy reserves on accomplishment of 3 factor security. In addition, the discussed protocols [3,19] consumes a lot of communication and computational overhead, therefore imposing a restriction on its practical realization for WSN-IoT resource constrained use cases.

Rao et al. [21] investigated and found that most of the devices on the internet are vulnerable to unsecured channel which may lead to exploitation of privacy. Authors have presented a light weight hashing method for user authentication in IoT. They proposed a customized BLAKE2b hashing algorithm with some modified elliptic curve digital signature scheme (ECDSA). The authors have compromised with the security in order to lessen the expenditure of communication and computation. The protocol is not verified for its resistance against the most basic attacks mentioned in Dolev-Yao [22] attack model, thus response of this scheme against attacks is very much unpredictable.

Das et al. [23] raised the concern of security and privacy of the information exchanged over the unsecured medium. Additionally, the authors discovered that existing schemes cannot be deployed in all use cases of IIoT due to their heavy computation and communication overheads. To overcome the aforementioned issues, the authors introduced a new privacy preserving user authentication scheme for IIoT environment. The scheme utilizes biometrics, fuzzy extractors, one way hash function, and XOR, and so forth to accomplish the authentication between the entities. The scheme makes use of smart card and biometrics together for verifying the authenticity of the entities. The authors have verified the robustness of their approach using Real or Random (RoR) model and declare their scheme as resistant against attacks. The proposed scheme is implemented in NS2 for operational performance evaluation. Despite the advantages, the scheme is energy expensive due to size and quantity of the messages exchanged during the authentication phase.

Li et al. [24] discussed the various applications where Wireless Sensor Networks (WSN) and IIoT are integrated to perform the tasks. The motivation of the authors to introduce a new security protocol is the threat of sensor node being accessed by an illegitimate user. The authors introduced the Elliptic Curve Cryptography (ECC) based authentication protocol for IIoT networks to overcome the deficiency of user anonymity and other vulnerabilities of the existing protocols. Their proposed algorithm used biometrics, fuzzy extractor, one way hash, and XOR for accomplishing the aim of authentication. The authors claimed their approach as secure based on the formal security analysis performed using the random oracle model. Post analysis, the scheme is simulated in NS3 (Network Simulator) to understand the behavior of the approach in the WSN-IoT environment. Regardless of the advantages, the scheme has not been tested against the potential attacks (e.g., MITM, etc.) that may prove fatal for the protocol. The scheme also lacks in ensuring message freshness and data confidentiality for all shared information due to absentia of nonce and ciphering mechanisms, respectively.

Paliwal in Reference [25] has highlighted the significance of WSN in gathering information from remote areas. The author emphasized the communication challenges due to the open nature of the channel and further advised the need for strong security protocols to preserve integrity and confidentiality. But it becomes a challenge in WSN, as the computing nodes have limited resources. The author has performed cryptanalysis on the existing techniques to prove the presence of vulnerabilities. A new mutual authentication and key exchange protocol has been proposed to address the deficits of the existing techniques. The new advised protocol exhibits various security features like integrity, conditional privacy, and so forth. The strength of the scheme against the attacks is analyzed using AVISPA (Automated Validation of Internet Security Protocols and Applications), RoR model, and informal analysis. The author claimed the scheme as robust and lightweight. Although the scheme exhibits various security attributes but still lacks in achieving overall privacy and identity anonymity. Besides the security attributes, substantial use of hash, and the large size of message overhead adds a burden on the overall scheme.

Chang et al. [26] demonstrated the weaknesses (e.g., inadequate secrecy, and impersonation attacks, etc.) of the existing security protocol through cryptanalysis. The authors introduced a new efficient and flexible smart card based authentication scheme for Ad hoc WSN to address the aforesaid security issues. The authors devised two protocols, wherein the Protocol $\left(P_{1}\right)$ overcomes the deficiencies of the existing protocol under scanner, and the Protocol $\left(P_{2}\right)$ provides secrecy to Ad hoc WSN with further modifications in $P_{1}$. However, the $P_{1}$ does not protect from all threats but lightweight, whereas, $P_{2}$ is stronger but resource expensive. Therefore, the selection of either protocol for the IIoT applications is a compromise. Despite the two solutions, the desire of a strong security protocol with limited computations remains unachieved.

Gope et al. [27] pointed out the various constraints (e.g., coverage, connectivity, and security, etc.) in the implementation of Industrial WSN (IWSN). The authors gave more importance to the security and privacy aspect as the information is exchanged over the unsecured medium with little human interventions. The real-time relaying of information in IWSN creates a requirement of a robust authentication protocol to protect the network from unauthorized abuses. The authors introduced a physically secure mutual authentication protocol for IWSN. The protocol uses hash, physically unclonable functions (PUF), and XOR as crypto primitives to accomplish the process of mutual authentication. The significant feature claimed by the authors is the protection of sensor nodes data even if it is physically captured by the adversary. The security features offered by the protocol are mutual authentication, and secure key exchange, and so forth. Nonetheless, the protocol exchange 6 large-sized messages during the authentication and key exchange phase. As IWSN make use of sensor nodes with limited resources, therefore deploying this protocol can reduce the active lifetime of the devices and network.

In the IoT domain, there are some standardization efforts to provide authenticated key exchange for constrained devices [28]; such as Ephemeral Diffie-Hellman Over COSE (EDHOC) [29] and Compact Transport Layer Security (CTLS)/Datagram Transport Layer Security (DTLS) v1.3 [30] handshake protocols. But it is investigated by the authors of Reference [31] that TLS (1.3) implementation errors can make the network vulnerable to Cache-like ATtacks (CATs), thus limiting its scope of utilization for sensitive networks for example, Industrial IoT. Also, the time sensitive information generated by manufacturing execution systems (SCADA, PLC, etc.) in Industrial IoT requires ultra low latency message exchanges. Therefore, industrial IoT network seeks a framework with limited number of layers and functionalities than TCP/IP [32]. Moreover, researchers argued that TCP/IP was originally developed for connecting mainframe computer to wired networks; therefore originally designed protocol stack of TCP/IP is not adequate enough to cater to the IoT architecture needs [33]. Internet Engineering Task Force (IETF) and researchers are trying to develop better alternatives. Due to inconclusive and inadequate standard architecture solutions, vendors are offering scalable, need specific (QoS, latency, etc.) and multipurpose IoT network architecture for private IIoT networks [34,35].

There are many alternatives proposed to TCP/IP protocol stack for Industrial IoT applications. Modbus Remote Terminal Unit (RTU) is an open standard used by the industries for performing the industrial tasks with critical timing requirements [36,37]. Similarly, Low powered wide area network (LPWAN) is recommended for Industrial networks (IIoT) because they are energy inexpensive and support long range communications [38,39,40,41,42,43,44]. Moreover, the latest version of LoRaWAN (v1.1) has shown to be secure against the vast majority of cyber-attacks and threats [45,46,47]. Hence, LoRaWAN is a secure and stable protocol that can be safely used not only for IoT applications but also for industrial ones. Various alliances and consortiums have been formed to build private LPWAN (open specifications) for various industrial applications, for example, DASH7 [48], LoRa [49], MIOTY [50,51], and Weightless [34], and so forth. DASH7, LoRa, and MIOTY, do not operate on TCP/IP stack rather operates on open protocol stack to provide flexibility to the IT administrator of the industry to choose the set of protocols based on application requirements (Quality of service (QoS), time sensitive, etc.) [52].

Recursive Internetwork Architecture (RINA) is also a good alternative to TCP/IP as it gives flexibility to manage the layered operations without interrupting the real time traffic. Moreover, RINA is claimed to be more secure than TCP/IP architecture as it secures the layers instead of protocols [53]. The review of available architectures [31,32,33,34,35,36,37,38,39,40,41,42,43,44,45,46,47,48,49,50,51,52,53] discloses the incompleteness of TCP/IP for IIoT networks and influence of open standards (LPWAN, RINA, etc.) for industrial applications. Apparently, the security protocols advised in TCP/IP stack may not be compatible with the open architectures, thereby opening vulnerabilities and posing threats to the security of the network. Conclusively, research is still in progress to develop the security protocols for open architecture based IIoT networks that can offer complete security solutions with limited utilization of resources.

It is important to note that security scheme proposed in this paper is specifically devised for open architecture based private IIoT networks.

### 2.1. Research Gaps

1. Most of the schemes studied in References [2,3,23,24,25,26,27] are found vulnerable to potential attacks.
2. Security features offered in References [2,3,23,24,25,26,27] are insufficient for sensitive IoT networks.
3. Traditional schemes [2,3,23,24,25,26,27] are computation and communication expensive.

### 2.2. Our Contribution

1. To encounter the threats, we have proposed a lightweight mutual authentication and key exchange protocol for IIoT (MAKE-IT).
2. To strengthen the security measures and achieve robustness, asymmetric and symmetric key cryptography have been used along with other crypto primitives such as hash and XOR, and so forth.
3. MAKE-IT exhibits various security features like data confidentiality, identity anonymity, mutual authentication, and prevention from unauthorized access, and so forth. Formal and informal analysis of MAKE-IT proves its capability to resist against these attacks: replay, modification, MITM, and impersonation attack.
4. MAKE-IT accomplishes mutual authentication and secret key exchange with high computational and communicational efficiency in comparison to existing schemes [2,3,23,24,25,26,27].

## 3. System and Adversary Model for Make-It Protocol

### 3.1. System Model

**Figure 3:**
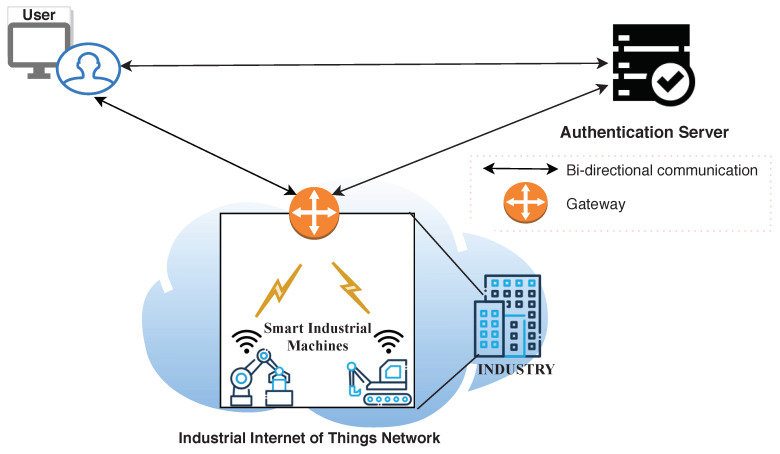
System model.

The system model describes the relationship between User, Gateway ($G_{W}$), Authentication Server (AS), and IIoT nodes. Figure 3 illustrates their relationship with each other.

#### 3.1.1. User

User could be industry manager, owner, administrator, and so forth who has privilege to control the machines, fetch the data from IIoT nodes, and so forth. User may access the network using any digital gadget like computer, laptop, and mobile, and so forth which has the capability to compute cryptography operations along with communication unit. User also requests the AS for generating the security credentials. Later, user utilizes the obtained credentials to generate the secured session key with the $G_{W}$.

#### 3.1.2. Gateway

Gateway ($G_{W}$) provides the interface to user for getting connected to the IIoT network. The gateways are not necessarily powered up through mains, rather depends upon the use case of IIoT. The present system model is constructed considering those applications of IIoT where $G_{W}$ is also a resource constrained node, for example, an industrial network deployed near volcano for monitoring eruptions, and extracting volcanic minerals, and so forth. $G_{W}$ receives partial security credentials of user from AS which is later utilized by $G_{W}$ to verify the legitimacy of the request. As communication to all the nodes of IIoT network is possible via $G_{W}$, any vulnerability in $G_{W}$ could compromise the whole network.

#### 3.1.3. Authentication Server (*AS*)

AS is a trusted entity whose prime responsibility is to validate the users and other devices of the network. It is assumed that user IDs are stored offline in the AS. The users request for the security credentials from AS. Upon being validated, AS provides a random secret integer to the user which is further utilized by the user to generate secret key with the gateway. AS is considered to be tamper proof entity and has no resource constraints.

#### 3.1.4. IIoT Nodes

Industrial machines are integrated with sensors (motion, proximity, vaccum, and pressure, etc.) and low power transceiver module (e.g., bluetooth, Zigbee, WiFi, etc.) for providing instant access to control and monitor the infrastructure of the industry. Legitimate users (manager, engineer, etc.) communicates to IIoT nodes via gateway. Note that this protocol is implemented to secure the network from external threats. Therefore, security considerations and message exchanges between user and gateway are only considered.

### 3.2. Adversary Model

MAKE-IT protocol has adopted the Dolev-Yao (DY) adversary model for evaluating the security performance under compromised conditions [22]. The threat model assumes that the attacker is competent enough to discover the vulnerabilities of the protocol; these vulnerabilities can be used by the attacker for mounting various attacks. Consider an industrial IoT network deployed near the volcano for monitoring eruptions and extracting volcanic minerals. Following the DY threat model, the user and the gateway (a network device communicating to autonomous mining machines, assembly lines, and driverless tipper trucks, etc.) are under threat in IIoT. Assume an attacker can eavesdrop all the communications happening between the user and the gateway. More precisely, an attacker can capture and replay the message for getting unauthorized access for example, machines, information, and so forth. In addition, an attacker can impersonate as an authorized user to steal precious information of volcanic minerals or locations where precious minerals are being kept. The adversary can try to modify the timestamp of the messages to get illegal access to the system to introduce malware for either disrupting or degrading the operations of the industry. The attacker can intercept the messages exchanged between the user and the gateway to extract the security parameters that are useful to approximate the future secret keys. The adversary can construct and inject new bogus messages to overwhelm the resources of the network device (gateway); as a result, the privileged user fails to deliver the messages to the gateway. Conclusively, the operational workflow of the industry will suffer and may result in financial and reputation loss.

## 4. Proposed Protocol: MAKE-IT

Assume a Industrial IoT environment where industrial machines are controlled and monitored over internet via gateway. Remote user can control and monitor the IIoT nodes after proving the legitimacy to gateway. Therefore, any vulnerability in authentication procedure could allow external attacker to access the network resources. In order to ensure legitimacy and avoidance of unauthorized access, we propose a light weight remote user authentication model. Note that in order to run the proposed protocol, we have assumed that gateway is resource constrained device and believe that all communications to industrial nodes happen via gateway. It is further assumed that clocks of all participating entities are synchronized to each other. The proposed protocol consist of two stages: User device registration phase, and Mutual Authentication & Secret Key Generation Phase.

### 4.1. User Device Registration Phase

Table 1 presents the notations that are used to describe the working of the protocol. Note that some Greek symbols have been used to represent variables; besides storing values, the symbols do not have any mathematical perspectives.

**Figure 4:**
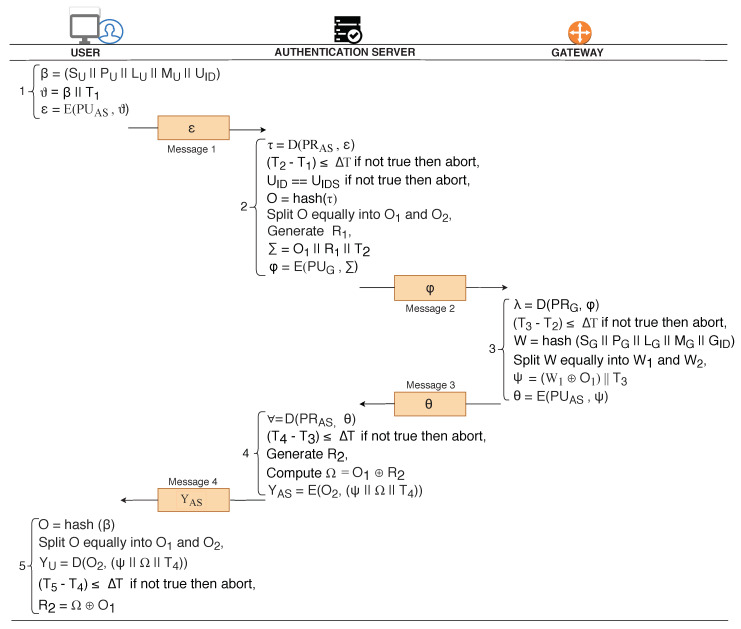
User device registration phase in MAKE-IT Protocol.

In this phase, User approaches Authentication Server (AS) to show his interest towards communication with Gateway. Users’ device initially prepares its identity details, β ($S_{U}$ || $P_{U}$ || $L_{U}$ || $M_{U}$ || $U_{ID}$). In addition, user adds a timestamp $\left(T_{1}\right)$ to message β, to prevent replay attacks. Finally user encrypts the message (β || $T_{1}$) and sends it to AS for obtaining security credentials (*random secret*). Figure 4 illustrates the complete process of user device registration with the AS.

AS receives the message (ϵ) from the user and decrypt it using private key, $D(PR_{AS}$, ϵ). AS verifies the lifetime of the request by compiling the timestamp values ($T_{2}-T_{1}$), fresh messages are processed and expired/replayed messages are discarded. Post timestamp verification, AS compares the received user identity, $U_{ID}$ with the offline stored user identity, $U_{IDS}$ for verifying the authenticity of the request; the session is aborted if comparison is not true. AS computes hash of decrypted message (τ) to preserve integrity. Further AS splits the hashed message (O) into two equal parts: $O_{1}$ and $O_{2}$. $O_{1}$ is utilized for processing of security credential request, whereas, $O_{2}$ is later utilized as a temporary key for securing the communications between AS and user. Afterwards, AS generates the random secret integer, $R_{1}$ and prepares the message (Σ = $O_{1}$ || $R_{1}$ || $T_{2})$ for gateway. Eventually Σ is encrypted with the public key of gateway, $E(PU_{G}$, Σ) to safeguard confidentiality. The encrypted message, $E(PU_{G}$, Σ) is then sent to $G_{W}$.

Gateway receives $E(PU_{G}$, Σ) from AS, decrypts using private key, $D(PR_{G})$ and forms λ. Before processing the message further, gateway verifies the timestamp of the message, $T_{3}-T_{2}$. Upon successful validation, gateway computes W = hash ($S_{G}$ || $P_{G}$ || $L_{G}$ || $M_{G}$ || $G_{ID}$). Gateway splits the hashed message (W) into two equal parts: $W_{1}$ and $W_{2}$. $W_{1}$ is utilized for processing of request, whereas, $W_{2}$ is later utilized to securely exchange the random secret of $G_{W}$ with the user. Further, gateway computes $W_{1}$⊕$O_{1}$ and concatenate timestamp $T_{3}$ to compose Ψ. Ψ is a useful component of protocol as it reflects a relationship between user and gateway. Additionally, Ψ is encrypted with the public key of AS, Θ = $E(PU_{AS}$, Ψ) to attain data confidentiality. The encrypted message (Θ) is forwarded to AS for further processing.

A.S decrypts the message $D(PR_{AS}$, Θ) using its private key. Upon successful decryption, freshness of the message is verified, $T_{4}-T_{3}$ followed by generation of random secret integer, $R_{2}$. AS computes Ω = $O_{1}$⊕$R_{2}$, assembles Ψ || Ω || $T_{4}$, and encrypts the assembled message to form $Y_{AS}$ = $E(O_{2},(\Psi$ || Ω || $T_{4}))$. The encrypted message $Y_{AS}$ is sent to the user. AS has utilized a secure mechanism to share the random secret with the user. As no one else know $O_{1}$, therefore only user is able to retrieve $R_{2}$.

User on the other hand, generates *O* by computing hash(β). The generated value *O* is splitted equally into $O_{1}$ and $O_{2}$. Using $O_{2}$, user decrypts the received message, $Y_{U}$ = $D(O_{2},(\Psi$ || Ω || $T_{4}))$. Post successful decryption, the user verifies the validity of the message, $T_{5}-T_{4}$. Upon verifying the genuineness, user computes the random secret integer, $R_{2}$ = Ω⊕$O_{1}$.

### 4.2. Mutual Authentication and Secret Key Generation Phase

In this phase, User and $G_{W}$ mutually verifies the legitimacy of each other before finalizing the secret key. User initially retrieves $W_{1}$ (= Ψ⊕$O_{1}$) and constructs $Z_{U}$ (= $E(W_{1}$, (Ω || $T_{5}$)). The random secret integer $R_{2}$ (enclosed in Ω) is securely shared with the gateway through $Z_{U}$.

**Figure 5:**
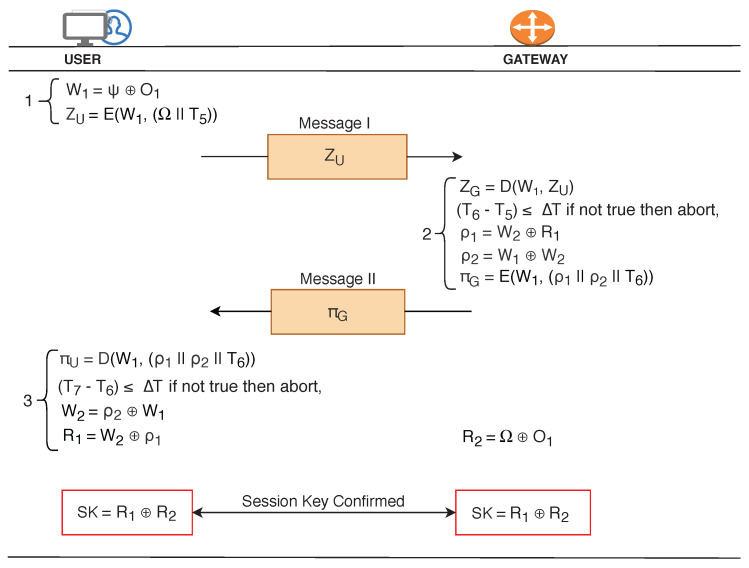
Mutual Authentication and Secret Key Generation Phase in MAKE-IT Protocol.

Gateway decrypts the received message, $Z_{G}$=$D(W_{1}$, $Z_{U})$ and verifies the timestamp, $T_{6}-T_{5}$. Timestamp verification shunts out the bogus (expired) and suspicious (replayed) requests. Post verification, gateway prepares $\rho_{1}$ and $\rho_{2}$ for hiding the random secret integer $R_{1}$, and parameter $W_{2}$, respectively. Subsequently, $G_{W}$ computes the $\pi_{G}$ $\{=E(W_{1}$, $(\rho_{1}$ || $\rho_{2}$ || $T_{6}))\}$, wherein $\rho_{1}$ carries hidden random secret integer $\left(R_{1}\right)$, $\rho_{2}$ carries hidden parameter value $\left(W_{2}\right)$ which is required by the user to retrieve the $R_{1}$ from $\rho_{1}$, and $T_{6}$ carries the present timestamp of the gateway. Thereafter, the encrypted message, $\pi_{G}$ is sent to the user. Figure 5 illustrates the whole process of mutual authentication and secret key generation phase.

The user decrypts the received message, $\pi_{U}$ $=D(W_{1}$, $(\rho_{1}$ || $\rho_{2}$ || $T_{6}))$ and verifies the time stamp, $T_{7}-T_{6}$. The connection is *either* terminated if the timestamp is stale *or* continues otherwise. Subsequently, user retrieves $W_{2}$ $(=\rho_{2}$⊕$W_{1})$. The retrieved $W_{2}$ is a key element required to recover the hidden random secret integer, $R_{1}$ $(=W_{2}$⊕$\rho_{1})$.

Successful decryption of $\pi_{U}$ and $Z_{G}$ results in mutual authentication between the user and $G_{W}$, respectively. Upon succeeding in mutual authentication, user and $G_{W}$ initiates the process of secret key formation. The secret keys are generated using random secrets ($R_{1}$ and $R_{2}$) issued by AS. User and $G_{W}$ have already exchanged the secret values required to form the secret key. Finally, user and the gateway computes the secret key, SK = $R_{1}$⊕$R_{2}$. The lifetime of the key depends upon the sensitivity of the application and may vary from few days to couple of months.

## 5. Security Analysis

The robustness of the proposed *MAKE-IT* approach is tested through security analyzer tool and informal analysis. This section demonstrates the test procedure and also presents the analysis of the test results.

### 5.1. Formal Analysis

Following References [54,55], the performance of the proposed protocol has been tested under the compromised conditions using AVISPA (Automated Validation of Internet Security Protocols and Applications). AVISPA is a security analyzer tool used to find vulnerabilities in the security protocols. It works on HLPSL (High Level Protocol Specification Language) and use an interpreter, *HLPSL*2*IF* which translates HLPSL to an Intermediate Format (IF). IF is presented as an input to the various back ends of AVISPA (e.g., on-the-fly model-checker (OFMC), Constraint-Logic-based ATtack SEarcher (CL-AtSe), etc.). The back ends compile the results and declare the protocol as *safe* or *unsafe*. We intentionally omitted the detailed discussion on the back ends of AVISPA, interested readers may refer to Reference [56].

The initial process is to script the subjected protocol into HLPSL language. The script begins with basic roles, followed by composition role, and ends with environment role. Basic roles declare the agents, crypto operations, compromised channel (dolev-yao), and various processes that are carried out locally by the agent. In contrast, composition roles declare the various legitimate entities that participate in the conversation. A very careful scripting of environment role is required as it may decide the effectiveness of this test. Environment role declares the global entities and constants. In addition, environment role describes the role and knowledge of intruder followed by various sessions that may exists during the communication. This role ends with the declaration of goals that defines the security attributes taken into consideration.

To assess the strength of the MAKE-IT protocol, the mutual authentication and key exchange phase has been scripted and examined on AVISPA. Note that notations used in HLPSL script is defined in Table 2.

**Figure 6:**
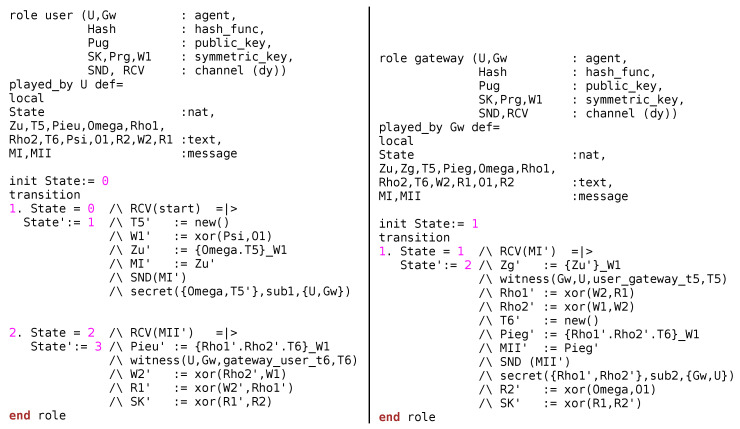
AVISPA Role Specification of the User and Gateway for our proposed MAKE-IT Protocol.

Initially basic roles of the *user* and $G_{W}$ are declared that comprises of local agents (*U*, $G_{W}$), crypto operations (hash), description of keys (SK, $PU_{G}$, etc.) and details of the compromised channel (*dy*) used for communication. Additionally, it describes the various local constants and messages used and exchanged during the conversation, respectively. User device gets activated in State = 0 (RCV(start)) whereas in State’:= 1 the user device generates a timestamp ($T5^{\prime }$), and computes $W1^{\prime }$. Afterwards, user computes $Zu^{\prime }$ = {Omega.T5}_W1 and forms the message, $MI^{\prime }$ (= $Zu^{\prime }$). The goal predicates set by the *user* is the privacy of the data (Omega and $T5^{\prime })$ along with the validation of the timestamp (T5) at Gw. The encrypted message ($MI^{\prime }$) is sent to the $G_{W}$ as shown in Figure 6.

$G_{W}$ receives the $MI^{\prime }$ in State = 1 and begins processing in State${}^{\prime }$:= 2. The foremost task performed by $G_{W}$ is the decryption of the received message, $Zg^{\prime }$ = {$Zu^{\prime }\}\_W1$. Post decryption, gateway validates the timestamp (witness(Gw,*U*,user_gateway_t5,T5)) to avoid replay attacks. Upon successful validation, $G_{W}$ computes $Rho1^{\prime }$ (= xor(W2,R1)) and $Rho2^{\prime }$ (= xor(W1,W2)). Subsequently, $G_{W}$ generates a fresh timestamp (T6), and computes $Pieg^{\prime }$ = $\{Rho1^{\prime }.Rho2^{\prime }.T6\}\_W1$ and compiles a message $MII^{\prime }$ (= $Pieg^{\prime }$). The goal predicates set by the Gw is the privacy of the data $(Rho1^{\prime }$ and $Rho2^{\prime })$ along with the validation of the timestamp (T6) at user. Thereafter, $G_{W}$ send the message to the user for extracting the required information to generate secret keys. Consequently, user decrypts the received message $\left(MII^{\prime }\right)$, $Pieu^{\prime }$ = $\{Rho1^{\prime }.Rho2^{\prime }.T6\}\_W1$. Post decryption, user validates the timestamp (witness(*U*,Gw,gateway_user_t6,T6)). Successful decryption of $Pieu^{\prime }$ and $Zg^{\prime }$ results in mutual authentication. Finally, user and $G_{W}$ use the retrieved information to generate the secret keys, SK.

Session role demonstrates the various constants, variables used by the entities during the communication for example, *User*(*U*,Gw,Hash,Pug,Prg,SK,W1,SU,RU), $G_{W}$(*U*,Gw,Hash,Pug,Prg,SK,W1, SGw,RGw). On the contrary, environment role is very prominent as it describes the constants and variables used globally by the agents. Furthermore, it describes the behaviour of the intruder{user, gateway,pug,prgi,ski,w1i,*h*}. Environment role also discusses the organizations of various sessions that may takes place between legitimate and illegitimate entities, for example, $Session_{1}$ (user,gateway,*h*,pug,prg,sk, w1), $Session_{2}$ (user,*i*,*h*,pug,prgi,ski,w1i), $Session_{3}$ (*i*,gateway,*h*,pug,prgi,ski,w1i).

**Figure 7:**
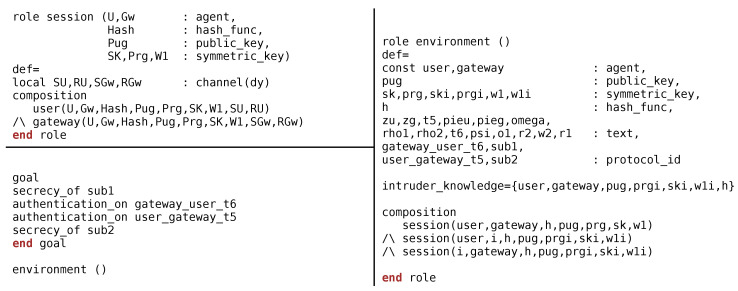
AVISPA Role Specification of the Session, Environment and Goal for MAKE-IT Protocol.

Finally, the environment role ends with declaration of goals of interest. The goals established to evaluate the robustness of proposed protocol is depicted in Figure 7 and listed here:

1. Secrecy_of sub1 represents that {Omega; T5} are kept secret between user and gateway.
2. Authentication_on gateway_user_t6 states that the timestamp (i.e., T6) of the message {$MII^{\prime }$} will be validated at the user.
3. Authentication_on user_gateway_t5 states that the timestamp (i.e., T5) of the message $MI^{\prime }$ will be validated at the $G_{W}$.
4. Secrecy_of sub2 represents that {Rho1; Rho2} are kept secret between gateway and user.

**Figure 8:**
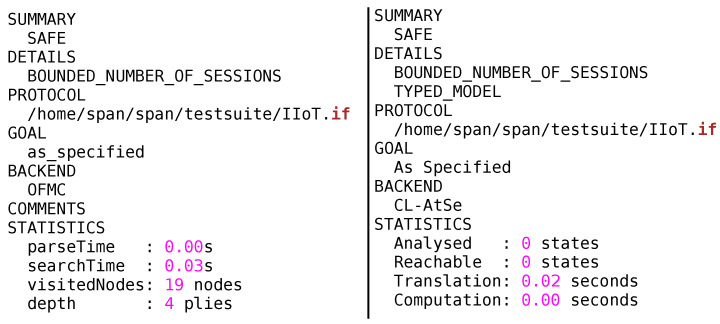
AVISPA results by using on-the-fly model-checker (OFMC) and Constraint-Logic-based ATtack SEarcher (CL-AtSe) backend for our proposed MAKE-IT Protocol.

MAKE-IT approach has been tested on two back ends of AVISPA that is, OFMC and CL-AtSe as illustrated in Figure 8. The Output file (OF) of OFMC and CL-AtSe backend clearly demonstrates that no vulnerability has been identified and the protocol is declared safe to use in Internet of Things applications. Conclusively, the protocol can withstand all the attacks mentioned in DY model while still maintaining the data privacy, authenticity and integrity of communications.

### 5.2. Informal Analysis

The informal security analysis of *MAKE-IT* approach has been discussed in this sub-section.

**Theorem** **1.** *Resistant to replay attacks.*

**Proof of Theorem** **1.** Freshness in each session is guaranteed as the messages ($M_{N}$) are composed of timestamps ($T_{N}$). $M_{1}$, $M_{2}$, $M_{3}$, $M_{4}$, $M_{I}$, and $M_{II}$, are all embedded with timestamps $T_{1}$, $T_{2}$, $T_{3}$, $T_{4}$, $T_{5}$, and $T_{6}$, respectively. Any misuse of expired message can be easily traced, for example, $T_{2}$ − $T_{1}$ ≤ ΔT. Assume an attacker eavesdropped the message, $M_{I}$ (Ω || $T_{5})$ and replay later to $G_{W}$ for getting unauthorized access. The $G_{W}$ receives the replayed message and decrypts, $D(W_{1}$, (Ω || $T_{5}{)}^{\prime }$. Post decryption, $G_{W}$ verifies the timestamp and analyse that received message contains old and expired timestamp, $T_{6}$ − $T_{5}$ > ΔT. The ΔT is usually kept very small to make it difficult for the adversary to replay the captured messages within ΔT. The $G_{W}$ instead of processing further discards the dishonest message. Additionally, the message $M_{I}$ is encrypted with the secret temporary session key $W_{1}$, hence making it computationally infeasible for the adversary to modify the timestamp $\left(T_{5}\right)$. Therefore, proposed protocol is resilient to replay attacks. □

**Theorem** **2.** *Resilient to man in the middle (MITM) attack.*

**Proof of Theorem** **2.** In MITM attack, adversary modifies the captured messages in such a way that destination cannot differentiate the modified message from the original message. Assume an attacker performs MITM attack between user and the gateway by capturing and modifying the message $M_{I}^{\prime }$ (= $E(W_{1}$, (Ω || $T_{5}{)}^{\prime })$. These computations are hard for attacker due to non availability of temporary secret key $\left(W_{1}\right)$ required for deciphering the captured message $D(W_{1}^{*}$, (Ω || $T_{5}))$ followed by ciphering of modified message $E(W_{1}^{*}$, (Ω || $T_{5}{)}^{\prime })$. Therefore, attacker fails to attempt MITM attack between the user and the gateway. Similarly, other messages $M_{1}$, $M_{2}$, $M_{3}$, $M_{4}$, and $M_{II}$ are also encrypted and hence cannot be modified. Therefore, the proposed scheme is protected from MITM attacks. □

**Theorem** **3.** *Secured against modification attack.*

**Proof of Theorem** **3.** Integrity is preserved due to use of one way hash function (i.e., SHA), for example, the element *O* = hash (τ) guarantees prevention against modification attacks. Any form of alterations in *O* can be easily identified during reconstruction and comparison of hash at other entity for example, $O_{1}^{\prime }$ == $O_{1}$. Apart from one way hash functions, the messages exchanged are encrypted to ensure that integrity of the communication is retained. Assume if attacker captures the message $M_{II}$ $\{=E(W_{1}$, $(\rho_{1}$ || $\rho_{2}$ || $T_{6}))\}$ and tries to modify $\{=E(W_{1}^{?}$, $(\rho_{1}$ || $\rho_{2}$ || $T_{6}{)}^{*})\}$. However, it is computationally difficult for the attacker to make any changes as the information is encrypted with the temporary secret key, $W_{1}$. Neither the key nor the security credentials (random secrets) are ever shared in plain text over the unsecured medium. Therefore, attacker does not find way to modify the content. Similarly, other messages $M_{1}$, $M_{2}$, $M_{3}$, $M_{4}$, and $M_{I}$ are ciphered to prevent modifications. Thus, proposed scheme is secured against modification attack. □

**Theorem** **4.** *Secure secret key generation.*

**Proof of Theorem** **4.** The proposed scheme ensures the secrecy during formation of secret key, SK. Secret key is formed using random secrets ($R_{1}$, $R_{2}$) generated by trusted and tamper proof entity, AS. User shares $R_{2}$ with gateway through message $M_{I}$ (= Ω || $T_{5})$. Likewise, gateway shares $R_{1}$ with user through $M_{II}$ $(=\rho_{1}$ || $\rho_{2}$ || $T_{6})$. Both $G_{W}$ and user retrieve $R_{2}$ & $R_{1}$ from $M_{I}$ and $M_{II}$, respectively. Finally, user and gateway generate a shared secret key, SK = $R_{1}$⊕$R_{2}$. As the keys are formed using random secrets which were never shared with anyone, therefore proposed protocol adheres to security measures while forming the secret key. The compliance to security measures ensures that secret key generated is not compromised and can be used for securing future communications. □

**Theorem** **5.** *Proposed scheme exhibits data confidentiality.*

**Proof of Theorem** **5.** Revealing of information to untrusted entities can pose serious threats to the existence of IIoT networks. Assume an attacker eavesdrop a message, $M_{3}$ $\left(=E\left(PU_{AS},\psi \right)\right)$. In spite of successful eavesdropping, the attacker would not be able to interpret the information due to the non availability of the private key of AS, $D(PR_{AS}^{?},\psi )$. The AS has never shared its private key $\left(PR_{AS}\right)$ with anyone, therefore, the attacker remains unsuccessful in obtaining the information from the captured message, $M_{3}$. In another instance, lets assume that attacker has captured, $M_{II}$ $\{=E(W_{1}$, $(\rho_{1}$ || $\rho_{2}$ || $T_{6}))\}$. The attacker has intentions to retrieve $W_{2}$ and $R_{1}$ from the captured message, $M_{II}$. Despite the successful capturing of $M_{II}$, the attacker would not be able to recover $W_{2}$ and $R_{1}$ from $\rho_{1}$ and $\rho_{2}$, respectively as the attacker needs a temporary secret key $\left(W_{1}\right)$ to decipher the information $\{=D(W_{1}^{?}$, $(\rho_{1}$ || $\rho_{2}$ || $T_{6}))\}$; the temporary secret key $\left(W_{1}\right)$ is shared amongst legitimate entities only. Similarly, the messages $M_{1}$, $M_{2}$, $M_{4}$, and $M_{I}$ are also encrypted, therefore, confidentiality of the information is ensured at all levels of communication. The attacker does not have these keys, $PR_{G}$, $PR_{AS}$, $O_{2}$, and $W_{1}$, to recover the overall information exchanged between the IIoT network entities. The proposed scheme exhibits the security property of data confidentiality. □

**Theorem** **6.** *MAKE-IT achieves identity anonymity.*

**Proof of Theorem** **6.** Identity anonymity is desirous to prevent the network from flooding based attacks, location aware attacks, and impersonation attacks, and so forth. MAKE-IT never discloses the identities of the network nodes to any unauthorized entity. Only AS has prepared an offline database of identities for verification purposes. AS is a trusted entity and stores the information in tamper proof memory, therefore any access or modification by the attacker is not possible. Even the parties involved in the communication does not know the real identities of each other, their identity details are hashed before being shared. Consider an attacker intercepted the message $M_{3}$ = $E(PU_{AS}$, Ψ) containing the hashed identity details of the $G_{W}$, still the attacker would not be able to interpret the identity due to hashing (W = hash ($S_{G}$ || $P_{G}$ || $L_{G}$ || $M_{G}$ || $G_{ID}$)) and ciphering of information, $E(PU_{AS})$. The attacker does not have the private key of AS $\left(PR_{AS}\right)$ to decipher the information. Therefore, MAKE-IT keeps the communication anonymous by not revealing the identities of user, gateway, and AS during the exchange of messages. □

## 6. Performance and Comparative Analysis

In this section, we evaluate the performance of the proposed protocol in terms of storage overhead, computational and communication cost. This section also presents the comparison analysis of proposed *MAKE-IT* approach with the traditional schemes [2,3,23,24,25,26,27] in terms of robustness against attacks and attainment of security features.

The storage cost requirement for implementing the proposed MAKE-IT approach is presented in Table 3. Total storage cost (all phases) of User, $G_{W}$ and AS are 264 bytes, 210 bytes, and 187 bytes, respectively. The storage space available in the CM5000 Telos B mote [57] (resource constrained device) is 1 MB, whereas the storage requirement to execute the proposed protocol (all phases) is just 0.02% of the total available memory space. The MAKE-IT approach achieves the goal of performing mutual authentication and key exchange with a small storage requirement. Apparently, the storage requirements are very nominal, thus making a way for its (MAKE-IT) applicability in all possible use cases of IIoT.

Table 4 demonstrates the computational cost spent by different entities (User, $G_{W}$ and AS) in all phases (user device registration, mutual authentication and key exchange) whereas Table 5 compares the computational cost requirements of proposed scheme with existing state of the art schemes. It can be clearly witnessed that the proposed scheme has less computations, thus imposing less burden on device processing, storage and battery resources. Note that we have only compared for mutual authentication and key exchange phase as the registration phase occurs once during initialization.

We have considered the Telos mote for calculating the communication cost of our scheme. Telos mote consumes 0.81 μJ and 0.72 μJ of energy for receiving and transmitting one bit, respectively [57]. Table 6 furnish the total communication cost spent by $G_{W}$ and *User* for performing mutual authentication and secret key generation. The results clearly signifies the efficiency of proposed scheme. The proposed protocol consumes only 385 μJ of energy whereas [2,3,23,24,25,26,27], consumes 768 μJ, 749 μJ, 658 μJ, 739 μJ, 742 μJ, 698 μJ, and 1411 μJ of energy, respectively. Therefore, existing schemes are not suitable for resource constrained environment of IIoT.

The robustness of the proposed protocol has been verified and presented in this section. The various security features offered by the proposed protocol accompanied with the list of attacks resisted by the protocol is presented in Table 7. It is observed from the Table that the proposed scheme exhibits strong protection against potential attacks and performs better in comparison to traditional schemes [2,3,23,24,25,26,27].

**Figure 9:**
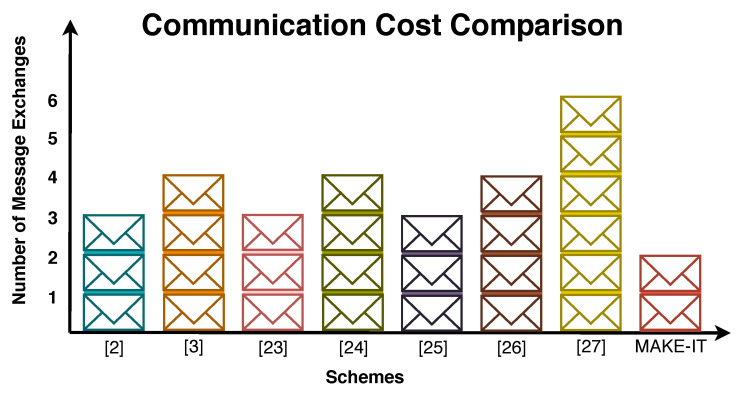
Communication Cost Comparison in terms of the number of message exchanges.

Figure 9 illustrates the number of messages exchanged by the resource constrained device during mutual authentication and key exchange phase. It is found during analysis that proposed scheme exchanges only 2 messages in comparison to 3, 4, 3, 4, 3, 4, and 6 messages of scheme [2,3,23,24,25,26,27], respectively. Less message exchanges in proposed scheme is a vital sign of efficient utilization of resources.

## 7. Conclusions & Future Scope

In this paper, we propose a lightweight remote user mutual authentication and key exchange model for IIoT. Industrial network can be protected from *external threats* if authenticity verification is performed before allowing any entity to access the network resources. The proposed scheme uses symmetric and asymmetric key cryptography, hash, timestamps, and so forth and various other crypto primitives to achieve secure mutual authentication and key exchange. The robustness of the scheme against attacks (replay attacks, modification attacks, and man in the middle attacks, etc.) is evaluated using formal and informal security analysis. The scheme proposed can withstand many popular attacks and offer many security features like data confidentiality, identity anonymity, integrity, and so forth. Further the proposed scheme is found to be resource efficient in terms of computation and communication. All these advantages of proposed protocol over existing schemes paves a path for its use in IIoT applications. The proposed scheme can further be extended in future to protect the industrial IoT networks from internal threats as well. Future work might also consider having a comparison of MAKE-IT protocol with TCP-UDP/IP security protocols under different network settings and parameters. Especially observing the performance comparison of our MAKE-IT protocol against others under the industrial network environment settings (where network delay is up most important) would be appealing.

## Figures and Tables

**Table 1:** Notations and Descriptions.

Notations	Description	Notations	Description
$U_{ID}$, $G_{ID}$	The user and gateway id	$R_{N}$	Random secret
$S_{U}$, $P_{U}$	Source and port address of user	$L_{U}$, $M_{U}$	Location and MAC address of user
$S_{G}$, $P_{G}$	Source and port address of gateway	$L_{G}$, $M_{G}$	Location and MAC address of gateway
SK	Shared secret key	$PR_{G}$, $PR_{AS}$	Private key of gateway and AS
h, ⊕	The hash and XOR operation	||	Concatenation operation
$M_{N}$, $T_{N}$	Messages and timestamps	$PU_{G}$, $PU_{AS}$	Public key of gateway and AS

**Table 2:** Notations and Descriptions for the symbols used in High Level Protocol Specification Language (HLPSL) script, Automated Validation of Internet Security Protocols and Applications (AVISPA).

Notations	Description	Notations	Description
*U*, *G_w_*	The *user* and *gateway*	*Pug, Prg*	Public and private key of gateway
*SK*	Secret key	*SND*, *RCV*	Compromised sending and receiving wireless channels
Pieu, Pieg	Alias $\pi_{U}$, $\pi_{G}$	.	Concatenation operation
Rho1, Rho2	Alias $\rho_{1}$, $\rho_{2}$	*dy*	dolev-yao attack model
Psi	Alias Ψ	{X}_Y	Encryption of component *X* with key, *Y*
*secret*, *witness*	Security goals	*SU, RU*	Sending and receiving channel of user
Omega	Alias Ω	*SGw, RGw*	Sending and receiving channel of gateway
*sub*1, *sub*2	Protocol ids	*i*	Intruder

**Table 3:** Storage cost of proposed protocol.

Parameter	Size (Bytes)	User	Authentication Server	Gateway
β, ν, $Y_{U}$, $Z_{U}$, $\pi_{U}$	16, 24, 48, 24, 40	✓		
τ, $U_{IDS}$, ∀, $Y_{AS}$, Σ	24, 1, 24, 48, 26		✓	
$O_{1}$, $O_{2}$	16, 16	✓	✓	
$R_{1}$, $R_{2}$	16, 16	✓	✓	✓
λ, $\rho_{1}$, $\pi_{G}$, Ψ, $Z_{G}$	26, 16, 40, 24, 24			✓
$W_{1}$, $W_{2}$, SK	16, 16, 16	✓		✓
**Total cost (bytes)**		264	187	210

**Table 4:** Computational cost of proposed protocol.

	Phase I	Phase II	Total cost
User	$C_{E}$ + $C_{D}$ + $C_{H}$ + $C_{XOR}$	$C_{E}$ + $C_{D}$ + $4*C_{XOR}$	$2*C_{E}$ + $2*C_{D}$ + $C_{H}$ + $5*C_{XOR}$
AS	$2*C_{E}$ + $2*C_{D}$ + $C_{H}$ + $2*C_{ran}$ + $C_{XOR}$	-	$2*C_{E}$ + $2*C_{D}$ + $C_{H}$ + $2*C_{ran}$ + $C_{XOR}$
Gateway	$C_{E}$ + $C_{D}$ + $C_{H}$ + $C_{XOR}$	$C_{E}$ + $C_{D}$ + $3*C_{XOR}$	$2*C_{E}$ + $2*C_{D}$ + $C_{H}$ + $4*C_{XOR}$
Total Cost	$4*C_{E}$ + $4*C_{D}$ + $3*C_{H}$ + $2*C_{ran}$ + $3*C_{XOR}$	$2*C_{E}$ + $2*C_{D}$ + $7*C_{XOR}$	$6*C_{E}$ + $6*C_{D}$ + $3*C_{H}$ + $2*C_{ran}$ + $10*C_{XOR}$

*Acronyms*: *C*: Computation, *E*: Encryption, *D*: Decryption, XOR: Ex-or operation, *H*: Hash, ran: random number generation, PhaseI: User device registration, PhaseII: Mutual authentication and secret key generation.

**Table 5:** Computation cost comparison with different schemes.

Schemes	Resource Constrained Device
[2]	$7*C_{H}$ + $C_{ran}$ + $6*C_{XOR}$
[3]	$4*C_{E}$ + $8*C_{H}$
[23]	$9*C_{H}$ + $5*C_{XOR}$
[24]	$9*C_{H}$ + $C_{ran}$ + $4*C_{XOR}$
[25]	$15*C_{H}$ + $10*C_{XOR}$
[26]	$10*C_{H}$ + $C_{XOR}$
[27]	$12*C_{H}$ + $2*C_{ran}$ + $8*C_{XOR}$
MAKE-IT	$C_{E}$ + $C_{D}$ + $4*C_{XOR}$

**Table 6:** Communication energy cost.

Schemes	Transmission (μJ)	Reception (μJ)	Total Energy Consumption (μJ)
[2]	459	309	768
[3]	229	520	749
[23]	371	287	658
[24]	348	391	739
[25]	$U_{D}$	742	742
[26]	279	419	698
[27]	371	1040	1411
MAKE-IT	230	155	385

*Acronyms*: $U_{D}$: Undisclosed.

**Table 7:** Analysis and Comparison of Protocols based on protection against attacks and security goals.

Attacks	[2]	[3]	[23]	[24]	[25]	[26]	[27]	MAKE-IT
Replay attack	✓	✓	✓	✓	✓	✓	✓	✓
Man in the middle attack	✓	×	✓	×	✓	×	✓	✓
Modification attack	✓	✓	✓	✓	✓	✓	✓	✓
Impersonation attack	✓	✓	✓	✓	✓	✓	✓	✓
Mutual authentication	✓	✓	✓	✓	✓	✓	✓	✓
Secure secret key	×	×	✓	✓	✓	✓	✓	✓
Prevention from unauthorized access	✓	✓	✓	✓	✓	✓	✓	✓
Data confidentiality	×	×	P	×	×	×	×	✓
Identity anonymity	P	✓	P	✓	P	P	✓	✓

*Acronyms*: ✓: Protected against attacks/Compliance to security goals, ×: Vulnerable against attacks/non compliance to security goals, P: Partially achieved.
